# Antitumor effects of anti-CD40/CpG immunotherapy combined with gemcitabine or 5- fluorouracil chemotherapy in the B16 melanoma model

**DOI:** 10.1186/2051-1426-1-S1-P86

**Published:** 2013-11-07

**Authors:** Alexander Rakhmilevich, Xiaoyi Qu, Mildred Felder, Zulmarie Perez Horta, Paul Sondel

**Affiliations:** 1 University of Wisconsin, Madison, WI, USA

## 

Our previous studies demonstrated that anti-CD40 mAb (anti-CD40) can synergize with CpG oligodeoxynucleotides (CpG) to mediate antitumor effects by activating myeloid cells, such as macrophages in tumor-bearing mice. Separate teams have shown that chemotherapy with gemcitabine (GEM) or 5-fluorouracil (5-FU) can reduce tumor-induced myeloid-derived suppressor cells (MDSC) in mice. In this study we asked if the same chemotherapy regimens with GEM or 5-FU will enhance the antitumor effect of anti-CD40 and CpG. Using the model of B16 melanoma growing intraperitoneally in syngeneic C57BL/6 mice, we show that these GEM or 5-FU treatment regimens either did not change or reduced, respectively, the number of MDSC in the peritoneal cavity of tumor-bearing mice. Treatment of mice with GEM or 5-FU did not significantly affect the antitumor function of macrophages as assessed in vitro. In vivo, treatment with these GEM or 5-FU regimens followed by anti-CD40/CpG resulted in antitumor effects similar to those of anti-CD40/CpG in the absence of GEM or 5-FU. Likewise, reduction of MDSC by in vivo anti-Gr-1 mAb treatment did not significantly affect anti-CD40/CpG antitumor responses. Together, the results show that the GEM or 5-FU chemotherapy regimens did not substantially affect the antitumor effects induced by anti-CD40/CpG immunotherapy.

